# Research on Compound PID Control Strategy Based on Input Feedforward and Dynamic Compensation Applied in Noncircular Turning

**DOI:** 10.3390/mi13020341

**Published:** 2022-02-21

**Authors:** Yong Zhang, Yue Huang, Yu Wang

**Affiliations:** 1 School of Advanced Manufacturing Technology, Guangdong Mechanical & Electrical College, Guangzhou 510550, China; zhangyong904@163.com; 2 State Key Laboratory of Digital Manufacturing Equipment & Technology, School of Mechanical Science and Engineering, Huazhong University of Science & Technology, Wuhan 430074, China; 2018010044@gdmec.edu.cn

**Keywords:** feedforward, PID, dynamic compensation, noncircular turning

## Abstract

The fast tool servo (FTS) control strategy is the control core of high-speed noncircular turning. This method should ensure high-speed and precision positioning and have the corresponding anti-interference ability in the micro-stroke motion with dynamic changes of tool feed and load. Most of the previous FTS control studies used the repetitive control and speed feedforward control strategy, which achieved promising results under ideal machining conditions. However, this strategy showed some defects in the real-world complex and changeable working conditions such as time-varying cutting force, intermittent cutting and fluctuating machine spindle speed. This paper proposed and implemented a compound proportional integral derivative control strategy based on input feedforward and dynamic compensation in noncircular turning. This technique successfully met the motion requirements of the high responsiveness of micro-stroke in noncircular turning and overcame disturbances from complex time variations of the cutting force, intermittent cutting case of the product and fluctuations of machine spindle speed. According to the findings, the machining tracking error was less than ±2 µm. Experimental results demonstrated the excellent tracking performance and machining effect of this control strategy.

## 1. Introduction

**Figure 1:**
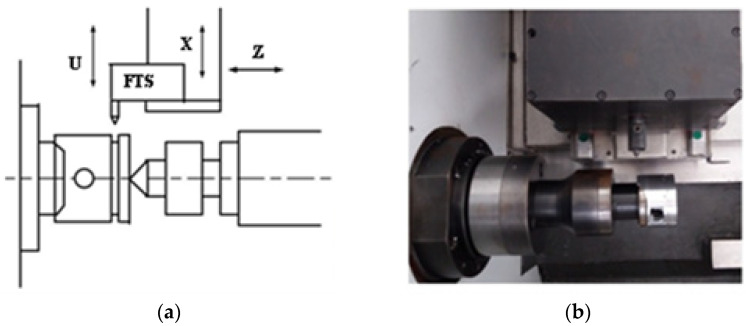
Fast tool servo structure. (**a**) Fast tool servo architecture; (**b**) Fast tool servo physical structure.

The motion characteristics of high-speed noncircular turning necessitate that the fast tool servo (FTS), which performs cutting feed, have high positioning accuracy and dynamic response performance. Importantly, the most challenging control criterion is that the FTS should not only ensure high speed and precision positioning but also have the anti-interference ability in the micro-stroke motion with dynamic changes of tool feed and load. Therefore, the control strategy of FTS in noncircular turning is crucial. Figure 1 shows the FTS structure. The FTS is equipped as the *U*-axis on the *X*-axis of a typical CNC lathe, with the *X*-axis performing the preliminary motion and the *U*-axis performing the high-frequency micro-motion that is synchronised with the spindle rotation and the motion of the *Z*-axis to cut the noncircular part.

Researchers have studied FTS in various ways. Some scholars proposed novel FTS mechanisms to facilitate noncircular turning. They developed a new piezoelectric actuator (PEA)-based FTS mechanism to incorporate additional functions to the general CNC system [1]. Different motor devices, such as noise voice coil motor and linear motor, among others, have also been studied as FTS drivers [2]. Some scholars investigated processing technology and parameter optimisation in noncircular turning to assess the processing performance of FTS [3,4]. In fact, all designs eventually require servo control, which emphasises the need for more control strategy research. Proportional integral derivative (PID) regulation is often used in traditional control strategies [5,6,7,8]. Many scholars have improved the basic PID principles in various applications [9,10,11]. Moreover, servo control directly affects the precision of non-circular machining; many studies have focused on the FTS control strategy [12]. For example, Zhang Y et al. proposed a PID controller based on feedforward and feedback PID control strategy; the controller was designed and embedded in the motion controller applied in noncircular piston turning [13]. Wang H et al. proposed some control solutions of fast tool servo in noncircular piston turning, they used new mechanisms as fast tool servo to meet requirements of turning [14]. Ma et al. presented a fast tool servo (FTS) system based on piezoelectric (PZT) voltage feedback and topology optimization, which was done to reduce the mass and compliance of the structure [15]. Mikalsen R et al. proposed the predictive piston motion control strategy in the free-piston internal combustion engine [16]. Wu D et al. designed repetitive control and feedforward control to obtain FTS frequency and load to meet noncircular machining requirements, with some of them considering the effects of velocity fluctuations [17].

Most previous FTS control studies based on various control strategies, such as speed feedforward control strategy, achieved good results under ideal machining conditions. However, when these strategies were applied to machining in actual working conditions, the machining effect was not ideal due to time-varying cutting forces, intermittent cutting and poor machining accuracy. After comparing the advantages and disadvantages of the speed feedforward control strategy, this paper proposed and applied a compound PID control strategy based on input feedforward and dynamic compensation in noncircular turning. This strategy successfully meets the motion requirements of the high response of micro-stroke in noncircular turning and overcomes disturbances from the time-varying complexities of the cutting force, the intermittent cutting case of the product and fluctuations in the machine spindle speed.

## 2. Mathematical Model of FTS

Figure 1 shows that the tool servo unit is used as the *U*-axis and mounted on the rough-positioned *X*-axis. Notably, the tool position along the *X*-axis must be properly synchronised with the spindle position for cutting the noncircular cross-section, which requires a high-speed, high-precision and high-frequency performance of the cutter control device. It is necessary to study the mathematical models of the motor and cutting force for effective control of the FTS.

### 2.1. Mathematical Model of the Motor

The mathematical model of the linear motor includes the voltage equation, the electromagnetic thrust equation and the mechanical motion equation.

**Figure 2:**
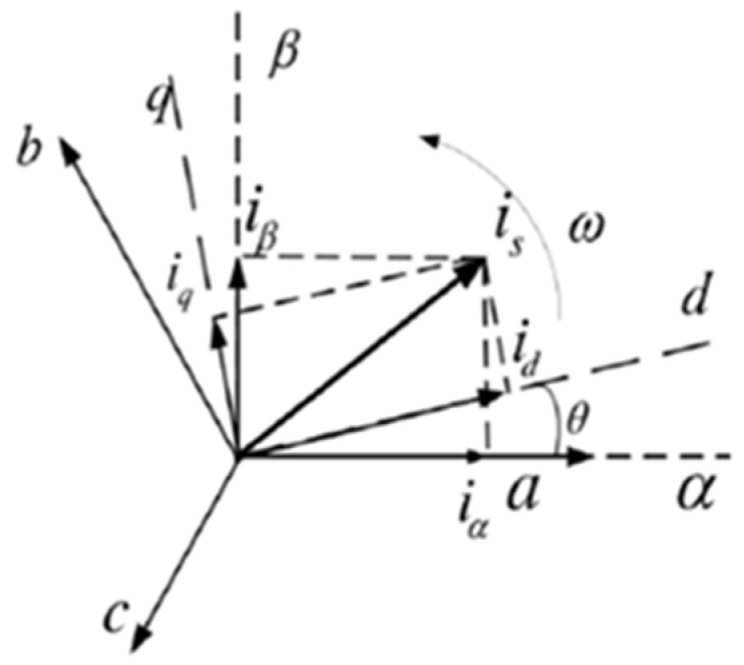
abc, α−β, d−q coordinate system.

A closer look at the motor structure reveals that a rotating motor can be referred to to construct a mathematical model of a permanent magnet synchronous linear motor. The specific idea is to use Clarke transformation to transform the system variable abc from the three-phase stationary coordinate system to the α−β two-phase stationary coordinate system according to the principle of coordinate transformation, and then use Park transformation to transform to the d−q two-phase synchronous rotating coordinate system. Finally, the mathematical relationship between output thrust and motor variables of the magnet synchronous motor (PMSM) is obtained [18,19]. Figure 2 shows the correspondence between the abc, α−β and d−q coordinate systems.

(1) Voltage equations for a linear motor

According to the conversion relation of the three coordinate systems shown in Figure 2 and the principle of vector control, the d−q voltage equation of the mathematical model of a permanent magnet synchronous linear motor can be expressed as follows [20]:

(1) $$\{\begin{array}{c} u_{d}=R_{s}i_{d}+L_{d}\frac{di_{d}}{dt}-\frac{\pi }{\tau }v\varphi_{q}\\ u_{q}=R_{s}i_{q}+L_{q}\frac{di_{q}}{dt}+\frac{\pi }{\tau }v\varphi_{d} \end{array}$$

where $u_{d}$ and $u_{q}$ represent the primary voltages of the d and q axes of the linear motor, respectively; $R_{S}$ is the equivalent resistance of a primary winding of the linear motor; $i_{d}$ and $i_{q}$ are the currents of the d and q axes, respectively; $L_{d}$ and $L_{q}$ are the inductances of the d and q axes, respectively; v is the speed of the linear motor; τ is the pole distance of permanent magnet of the linear motor; and $\varphi_{d}$ and $\varphi_{q}$ are the flux chains of the d and q axes, respectively, whose flux formula is shown in the following equation.

(2) $$\{\begin{array}{c} \varphi_{d}=L_{d}i_{d}+\varphi_{f}\\ \varphi_{q}=L_{q}i_{q}\;\;\;\;\;\;\;\;\;\; \end{array}$$

where $\varphi_{f}$ is the permanent magnet excitation fundamental flux chain of the linear motor.

(2) Electromagnetic thrust equation of the linear motor

The electromagnetic thrust equation of a permanent magnet synchronous linear motor can be expressed as follows:

(3) $$F_{e}=K\left[\varphi_{f}i_{q}+\left(L_{d}-L_{q}\right)i_{d}i_{q}\right]$$

where $F_{e}$ is the electromagnetic thrust of the linear motor and $K=\frac{3\pi }{2\tau }$ is the inverse electric system constant of the linear motor.

Ideally, the inductances of the d and q axes could be considered equal, $L_{d}=L_{q}=L$. Hence, Equation (3) could be converted into Equation (4) as follows:

(4) $$F_{e}=K_{f}i_{q}$$

where $K_{f}=\frac{3\pi }{2\tau }\psi_{f}$ is the electromagnetic thrust coefficient of the linear motor.

(3) Mechanical motion equation of the linear motor

According to the analysis of the motion force of the linear motor, the mechanical motion equation can be expressed as follows:

(5) $$m_{s}\frac{dv}{dt}+Bv+F_{l}=F_{e}$$

where $m_{s}$ is the moving mass of the linear motor, v is the speed of the linear motor, B is the friction coefficient, $F_{l}$ is load resistance and $F_{e}$ is the electromagnetic thrust of the linear motor.

To sum up, Equations (1), (4) and (5) constitute a brief mathematical model of the permanent magnet synchronous linear motor.

**Figure 3:**
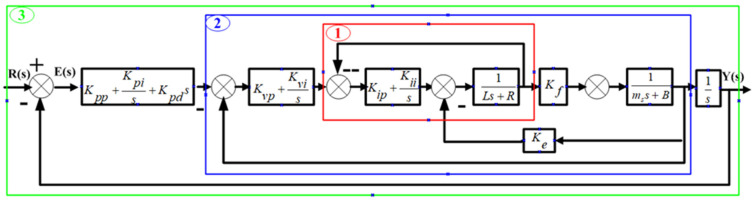
Control diagram of fast tool servo system. 1—current loop; 2—speed loop; 3—position loop.

The FTS system is a closed-loop system with position feedback driven by a linear motor. The control system uses a three-ring control structure of position, speed and current, with PID/PI/PI as the control mode, respectively. The basic PID control model of the FTS can be obtained via the linear motor mathematical model, as in Figure 3.

In Figure 3, $K_{pp}$ is a scale factor, $K_{pi}$ is an integral coefficient, $K_{pd}$ is a differential coefficient, $K_{vp}$ is a scale coefficient, $K_{vi}$ is an integral coefficient and $K_{ip}$ is a proportional coefficient. Furthermore, R(s) and Y(s) are the input and output signals of the system, respectively, and E(s) is the deviation signal of the system. The stability of the system and the nature of dynamic response can be proved by analysing the dynamic characteristics of current, velocity and position rings.

### 2.2. Mathematical Model of the Cutting Force

**Figure 4:**
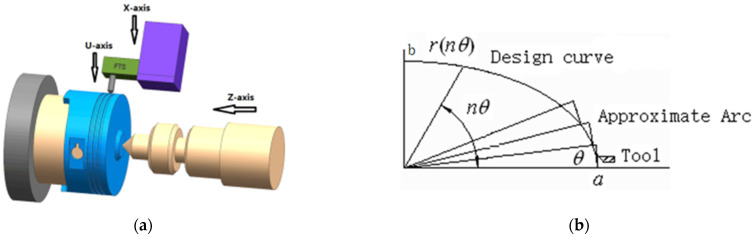
Feed analysis of non-circular turning tool. (**a**) Multi-axis feed for non-circular turning; (**b**) Fast tool servo micro feed.

Because the cross-section of the noncircular part is usually elliptical, its high-frequency noncircular turning uses the multi-axis control feed mode, as in Figure 4a. Figure 4a shows that FTS micro feed is performed for every differential resolution angle according to the real-time feedback angle of the spindle encoder. The tool trajectory approximates the noncircular contour with a segment of tiny arc (Figure 4b).

The equation of the polar diameter R with an angle of θ on elliptical contour is shown as follows [21]:

(6) $$R=\sqrt{a^{2}\cos^{2}\theta +b^{2}\sin^{2}\theta }$$

In Equation (6), θ∈[0,2π], a is the length of the long axis, b is the length of the short axis. Equation (6) can be transformed into time-based equations of motion as follows:

(7) $$R=\sqrt{a^{2}\cos^{2}(2\pi nt)+b^{2}\sin^{2}(2\pi nt)}$$

where n is the speed of the spindle.

The tool’s starting position is set at the long axis vertex of the elliptical contour and its initial value is set to zero. The motion trajectory equation of the tool can be deduced as follows:

(8) $$\begin{array}{c} x=a-R\\ =a-\sqrt{{\left(a\cos(2\pi nt)\right)}^{2}+{\left(b\sin(2\pi nt)\right)}^{2}} \end{array}$$

Based on Equation (8), the velocity and acceleration mathematical model of the tool can be calculated further.

The turning force of the noncircular surface part is the main interference force, which directly affects the motion control of the cutting tool. In noncircular turning, the cutting force of the tool is uneven and dynamic. However, the force on a static point is similar to that on a normal circular section, which contains tangential force $F_{c}$, radial force $F_{p}$ and axial force $F_{f}$. The exponential empirical equations for cutting forces are as follows:

(9) $$\{\begin{array}{c} F_{c}=K_{Fc}\cdot a_{p}^{xFc}\cdot f^{yFc}\cdot {\left(60v_{c}\right)}^{nFc}\\ F_{p}=K_{Fp}\cdot a_{p}^{xFp}\cdot f^{yFp}\cdot {\left(60v_{c}\right)}^{nFp}\\ F_{f}=K_{Ff}\cdot a_{p}^{xFf}\cdot f^{yFf}\cdot {\left(60v_{c}\right)}^{nFf} \end{array}$$

where $K_{Fc}$, $K_{Fp}$ and $K_{Ff}$ are the influence coefficients of the workpiece material on the cutting force; $xF_{c}$, $xF_{p}$ and $xF_{f}$ are the influence coefficients of back feed on the cutting force; $yF_{c}$, $yF_{p}$ and $yF_{f}$ are the influence coefficients of feed on the cutting force; $nF_{c}$, $nF_{p}$ and $nF_{f}$ are the influence coefficients of cutting speed on the cutting force; $a_{p}$ is the back engagement of the cutting edge (mm); f is feed (mm/r); $v_{c}$ is the cutting speed (m/s).

Equation (9) shows that, although the force analysis of noncircular turning is the same as that of the normal turning at a particular moment, the $a_{p}$ and $v_{c}$ values of each elliptical cross-section are constantly changing in the actual machining process, meaning that the cutting force is also constantly changing. Equation (9) can be converted into Equation (10), which is based on time t. For noncircular turning, the radial force $F_{p}$ acts directly on the radial motion of the tool, which is the main interference force. Therefore, the electromagnetic driving force of the FTS system should be greater than the sum of the radial partial force $F_{p}$ and the moving inertial force when the radial friction force is ignored. The force equation is shown as follows:

(10) $$\begin{array}{c} F=F_{p}+ma\\ \begin{array}{l} =K_{Fp}\cdot {\left(\frac{a-b}{2}(1-\cos4\pi nt)\right)}^{xFp}\cdot f^{yFp}\cdot \\ {\left(60(2\pi (r-\frac{a-b}{2}(1-\cos4\pi nt))n/1000)\right)}^{nFp} \end{array}\\ \begin{array}{l} +m(\frac{4\pi^{2}n^{2}(a^{2}-b^{2})\cos(4\pi nt)}{R}+\\ \frac{\pi^{2}n^{2}{\left(a^{2}-b^{2}\right)}^{2}\sin^{2}(4\pi nt)}{R})/1000 \end{array} \end{array}$$

where m is the mass of the moving part of the noncircular feed system and n is the speed of the spindle.

## 3. Compound PID Control Strategy Based on Speed/Acceleration Feedforward

### 3.1. Compound Feedforward PID Control Principle

**Figure 5:**
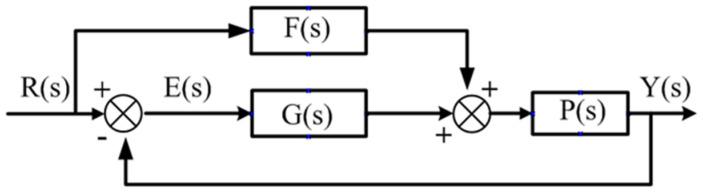
Compound feedforward PID control structure.

Many researchers combined feedforward and PID control to solve the problem of control lag and improve the tracking effect of the system on the input signal. Figure 5 shows the structure of the compound feedforward PID control in which F(s) is the transfer function of the feedforward control, G(s) is the transfer function of the PID feedback control, P(s) is the transfer function of the control object, R(s) is the input signal, Y(s) is the output signal and E(s) is the deviation of the input and output signals.

The closed-loop transfer function of compound feedforward PID control can be expressed as follows:

(11) $$G_{b}(s)=\frac{Y(s)}{R(s)}=\frac{P(s)(F(s)+G(s))}{1+G(s)P(s)}$$

The transfer function of the system control deviation E(s) can be expressed as follows:

(12) $$E(s)=\frac{1-F(s)P(s)}{1+G(s)P(s)}R(s)$$

It is necessary to make the control deviation E(s)=0 to achieve no lag between the output and input of the system.

### 3.2. Application of Velocity/Acceleration Feedforward PID Control in Noncircular Turning

The compensation signal of the speed feedforward is used for improving the response precision of system speed in the control of FTS based on the combination of PID control and speed and acceleration feedforward. The compensation signal of the acceleration feedforward can effectively suppress the overshoot caused by the speed feedforward control.

**Figure 6:**
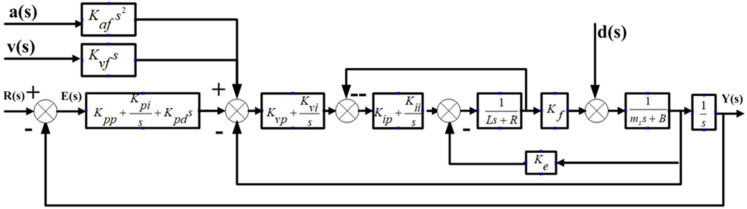
Speed/acceleration feedforward controller model.

Figure 6 shows the controller model for speed and acceleration feedforward, in which $K_{vf}$ is the speed feedforward and $K_{af}$ is the acceleration feedforward.

**Figure 7:**
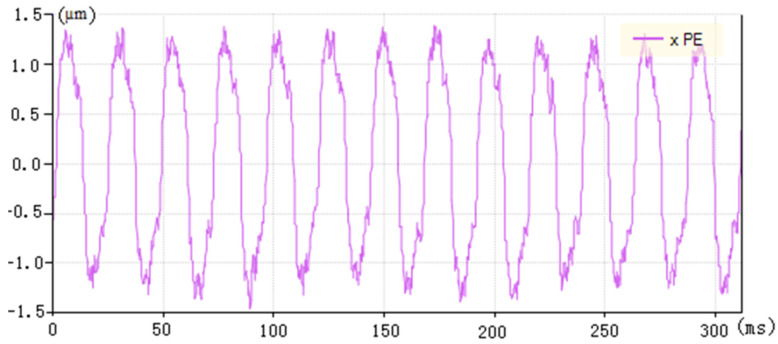
Tracking error of velocity and acceleration feedforward control under no-load machining (xPE is the abbreviation of tracking error).

The PID control strategy of velocity and acceleration feedforward can achieve a good control effect when there is no load and no consideration of cutting force. Figure 7 shows a waveform diagram of the tracking error under no load machining, with a feed speed of 240 mm/min, a spindle speed of 1200 rpm, an ellipse of 0.5 mm and a profile of 0.1 mm. The maximum tracking error is ±1.3 μm.

**Figure 8:**
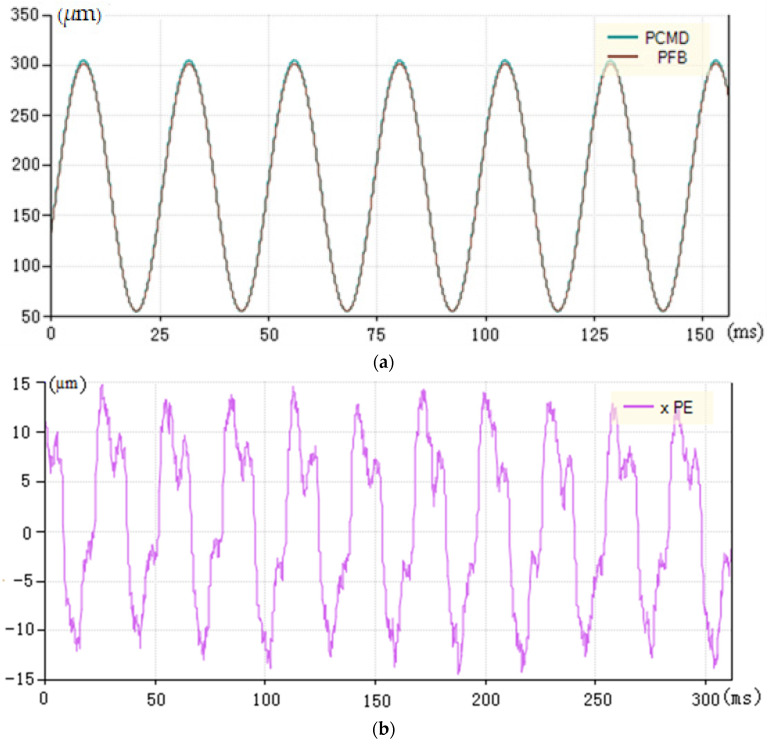
Position curve and tracking error of the speed and acceleration feedforward control under load processing. (**a**) Instruction and feedback curve under load processing (PCMD is the abbreviation of instruction position; PFB is the abbreviation of feedback position). (**b**) Tracking error under load processing (xPE is the abbreviation of tracking error).

However, the cutting force acting as the main interference constantly changes in the actual turning process. As a result, the PID control strategy of velocity and acceleration feedforward cannot effectively deal with these disturbances. Figure 8a shows that the tracking performance of the arc top deteriorated. The tracking error map of Figure 8b shows that the maximum tracking error is up to 14 µm, with a significant fluctuation in the tracking error at the peak.

The above experiment analysis shows some nonlinear factors, such as time-varying cutting force and broken cutting, among others, in the noncircular turning process. The PID control strategy with speed and acceleration feedforward is not ideal under load machining; hence, the nonlinear factors in noncircular turning must be considered and solved.

## 4. Compound PID Control Strategy Based on Input Feedforward and Dynamic Compensation

Compound feedforward can be divided into feedforward control that is compensated by input and feedforward control that is compensated by disturbance [22]. The input compensation can improve the ability and precision of the system to reproduce the input signal and reduce the steady-state error. The disturbance compensation can suppress the effects of various measured disturbance signals and improve the anti-interference ability and robustness of the system.

The input compensation feedforward control with speed feedforward and acceleration feedforward can achieve a good control effect without considering disturbance in high-speed noncircular turning. In contrast, the cutting tool’s most important disturbance signal (cutting force) can be used for the feedforward control of disturbance compensation according to the pre-calculated dynamic model. Therefore, the compound PID control strategy based on input feedforward and dynamic compensation can achieve a good tracking control effect in high-speed noncircular turning while also meeting the requirements of high response motion control of high-speed noncircular turning.

### 4.1. Perturbation Model

**Figure 9:**
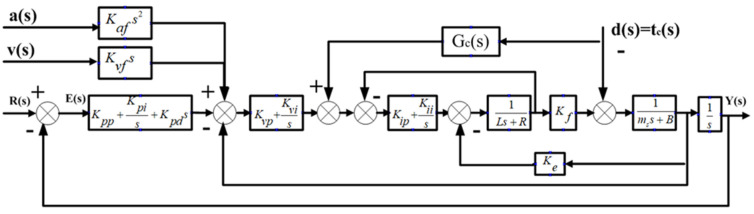
Controller model of input feedforward and disturbance compensation.

**Figure 10:**
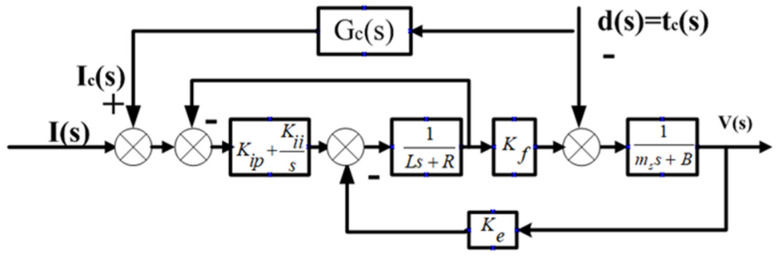
Disturbance feedforward compensation based on dynamic model.

In the noncircular turning process, the cutting force disturbance is compensated on the current ring of the PID controller to achieve a more rapid and effective control effect based on the original input feedforward compensation. Figure 9 shows the overall model of the controller. Figure 10 shows the disturbance compensation model.

As depicted in Figure 10, the transfer function $G_{dy}\left(s\right)$ based on the estimable disturbance $t_{c}\left(s\right)$ and the output V(s) is expressed as follows:

(13) $$G_{dy}(s)=\frac{V(s)}{t_{c}(s)}=\frac{G_{c}(s)K_{f}K_{I}-K_{I}-(Ls^{2}+Rs)}{(ms+B)(Ls^{2}+Rs+K_{I})+K_{f}K_{e}s}$$

where $K_{I}=K_{ip}s+K_{ii}$. The transfer function $G_{dy}\left(s\right)$ must be zero to compensate for the disturbance $t_{c}\left(s\right)$ in the system processing. For Equation (13), the molecule can be set to zero and the following equation can be obtained:

(14) $$G_{c}(s)K_{f}K_{I}-K_{I}-(Ls^{2}+Rs)=0$$

According to Equation (14), the transfer function $G_{c}\left(s\right)$ from $t_{c}\left(s\right)$ to the disturbance compensation output equivalent current $I_{c}\left(s\right)$ can be obtained as follows:

(15) $$G_{c}(s)=\frac{t_{c}(s)}{I_{c}(s)}=\frac{K_{I}+(Ls^{2}+Rs)}{K_{f}K_{I}}=\frac{1}{K_{f}}+\frac{Ls^{2}+Rs}{K_{f}[K_{ip}s+K_{ii}]}$$

At the same time, based on the analysis of cutting force in Section 2.2, the formula of cutting force is expressed as follows:

(16) $$F_{p}=K_{Fp}\cdot x^{xFp}\cdot f^{yFp}\cdot {\left(60(2\pi (r-x)n/1000)\right)}^{nFp}$$

where $K_{Fp}$ is the influence coefficient of the material of the tool and the material of the workpiece on the cutting force; xFp is the influence coefficient of the back feed; yFp is the influence coefficient of main feed; nFp is the influence coefficient of cutting speed on the cutting force; x is the cutter quantity (mm); f is the feed speed (mm/r); r is the workpiece turning radius (mm); and n is the spindle speed (rpm).

This paper only studied the cutting force of noncircular turning, which is expressed as follows:

(17) $$\begin{array}{l} d(s)=t_{c}(s)=K_{Fp}\cdot x{\left(s\right)}^{xFp}\cdot f^{yFp}\\ \cdot {\left(60(2\pi (r-x(s))n/1000)\right)}^{nFp} \end{array}$$

If the workpiece is a middle convex variable elliptical piston, then Equation (17) can be further expressed as follows:

(18) $$\begin{array}{l} d(s)=K_{Fp}\cdot {\left(\frac{a-b}{2}(1-\cos4\pi nt)\right)}^{xFp}\cdot f^{yFp}\\ \cdot {\left(60(2\pi (r-\frac{a-b}{2}(1-\cos4\pi nt))n/1000)\right)}^{nFp} \end{array}$$

where a is the long axis of the cross-section ellipse and b is the minor axis of the cross-section ellipse.

### 4.2. Application of Compound PID Control with Input Feedforward and Dynamic Compensation in High-Speed Noncircular Turning

**Figure 11:**
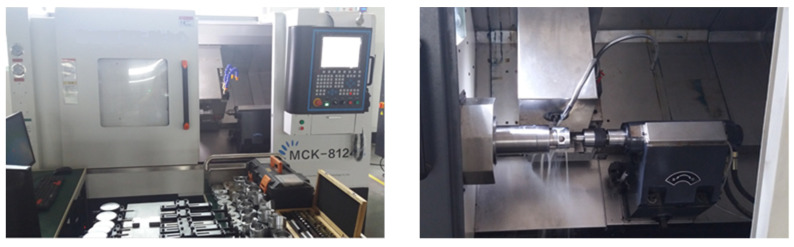
Machine tool.

This section details the machining test based on the compound PID control strategy of input feedforward and dynamic compensation. The dynamic prospective compensation is directly compensated before the current loop of the servo driver after real-time calculation. In the experiment, the middle convex variable elliptical piston of aluminium alloy is taken as the machining object whose main parameters are shown in Table 1. Figure 11 shows the machine tool that performs the control strategy in its control system.

**Figure 12:**
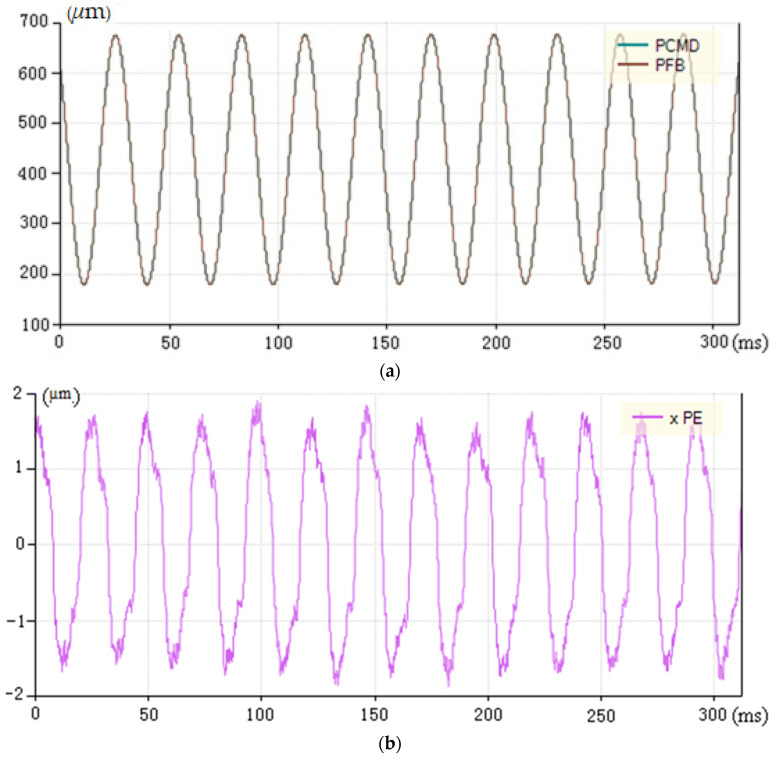
Position curve and tracking error based on compound PID control of feedforward and dynamics compensation under load machining. (**a**) Position and feedback curve of compound PID control based on input feedforward and dynamic prospective compensation (PCMD is the abbreviation of instruction position, PFB is the abbreviation of feedback position). (**b**) Tracking error curve based on compound PID control of input feedforward and dynamic prospective compensation (xPE is the abbreviation of tracking error).

Figure 12a shows the curve of instruction position and feedback position of load machining that used the compound PID control of input feedforward and dynamic prospective compensation with overall good tracking performance. Figure 12b shows the tracking error curve of load machining in this control mode. The maximum tracking error is ±1.9 μm, which is much better than the previous tracking error used in the control of speed and acceleration feedforward PID.

**Figure 13:**
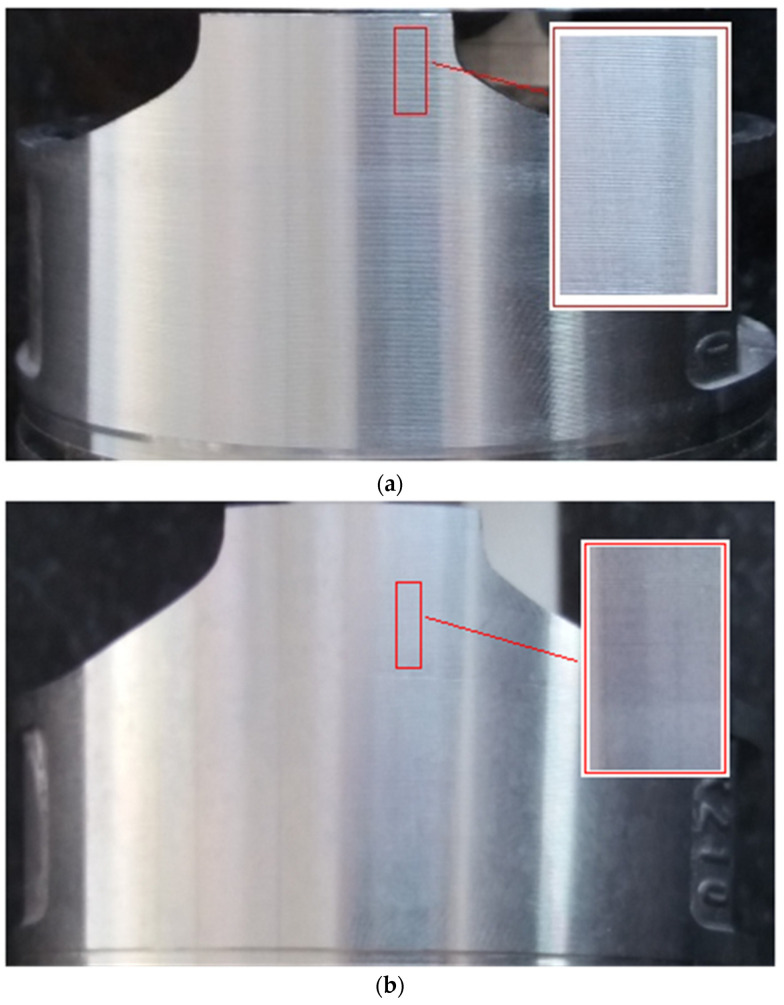
Comparison of Surface patterns machined by different control methods. (**a**) Piston surface controlled by speed and acceleration feedforward compensation. (**b**) Piston surface controlled by compound PID of input feedforward and dynamic compensation.

The compound PID control strategy with input feedforward and dynamic compensation can effectively improve the tracking performance of machining under complex and changeable noncircular turning conditions. Figure 13 shows the improvement of the surface pattern of the piston machined by experiment. Figure 13a shows the piston surface machined by the speed and acceleration feedforward PID control. The tracking error is too large to reach ±14 µm, the surface roughness is 1.01 µm and the depth and the shallow consistency of piston skirt are poor. Figure 13b shows the piston machined by the compound PID control of input feedforward and dynamic prospective compensation. The tracking error is less than ±2 μm, the surface roughness is 0.61 µm and the depth and the shallow consistency of piston skirt are of high quality. Furthermore, the experimental processing of other speed and size parameters shows that the control strategy is equally superior, Table 2 shows processing results of different speed parameters, Table 3 shows processing results of different ovality and profile parameters.

## 5. Conclusions

The control strategy of an FTS is the control core of high-speed noncircular turning. Based on the mathematical model of FTS and a comparison of the advantages and disadvantages of the repetitive control and the speed feedforward PID control in noncircular turning, a compound PID control strategy based on input feedforward and dynamic compensation is proposed in this paper to meet the motion characteristics of high-speed noncircular turning with high response and micro-stroke. The main contributions of this study are summarised as follows:

(1) The mathematical model of the FTS is proposed to investigate the control method and cutting force. The PID control model of the FTS is derived from the analysis of a mathematical model of a linear motor that includes voltage equation, electromagnetic thrust equation and mechanical motion equation. The force analysis shows that the cutting force is constantly changing because of the changes in speed and acceleration. It can be concluded that noncircular turning is more complex than ordinary turning.
(2) The test results show that the machining tracking error is improved from ±14 µm to less than ±2 µm by using the compound PID control strategy based on input feedforward and dynamic compensation. The depth and shallow consistency of the piston skirt are neat and compliant. The findings demonstrate that this strategy fully considers the time-varying complexity of the actual cutting force, the diversity of intermittent cutting and fluctuations in the machine spindle speed. The experimental results also demonstrate that the strategy has a very good tracking performance and machining effect.

## Figures and Tables

**Table 1:** Experiment parameters of cutting force and machining parameters.

Name	Parameter
material	aluminum alloy
$K_{Fp}$	606.36
xFp	0.76
yFp	0.7
nFp	0.04
S (spindle speed (rpm))	1500
F (Z feed (mm/min))	250
maximum ovality (mm)	1
maximum profile (mm)	0.5

**Table 2:** Processing results of different speed parameters.

Name	1	2	3	4	5
S (spindle speed (rpm))	1500	1350	1200	1050	900
F (Z feed (mm/min))	250	225	200	175	150
maximum ovality (mm)	1	1	1	1	1
maximum profile (mm)	0.5	0.5	0.5	0.5	0.5
tracking error (μm) (general control)	14	13.5	13.2	13	12.9
tracking error (μm) (compound PID control)	2	2	1.9	1.8	1.8
surface roughness (μm) (general control)	1.01	0.96	0.96	0.92	0.91
surface roughness (μm) (compound PID control)	0.61	0.58	0.58	0.57	0.56

**Table 3:** Processing results of different ovality and profile parameters.

Name	1	2	3	4	5
S (spindle speed (rpm))	1500	1500	1500	1500	1500
F (Z feed (mm/min))	250	250	250	250	250
maximum ovality (mm)	1	0.9	0.8	0.7	0.6
maximum profile (mm)	0.5	0.4	0.3	0.2	0.1
tracking error (μm) (general control)	14	13.8	13.7	13.5	13.5
tracking error (μm) (compound PID control)	2	2.05	2	1.9	1.9
surface roughness (μm) (general control)	1.01	0.95	0.92	0.91	0.91
surface roughness (μm) (compound PID control)	0.61	0.60	0.60	0.58	0.58
